# P-1484. How do recent population-level RSVpreF vaccine impact results from the United Kingdom relate to previously reported vaccine effectiveness results?

**DOI:** 10.1093/ofid/ofaf695.1669

**Published:** 2026-01-11

**Authors:** Negar Aliabadi, Qing Liu, Carmen Hockey, Andrew Vyse, Gillian Ellsbury, Luis Jodar, Elizabeth Begier

**Affiliations:** Pfizer, New York, NY; Pfizer Inc., Collegeville, Pennsylvania; Pfizer, New York, NY; Pfizer UK, Tadworth, England, United Kingdom; Pfizer ltd, London, England, United Kingdom; Pfizer Vaccines, Collegeville, Pennsylvania; Pfizer Vaccines, Collegeville, Pennsylvania

## Abstract

**Background:**

The United Kingdom (UK) introduced RSVpreF vaccine into its national immunization program for persons 75 years of age (with catch up for those 76–79 years) in 2024, first in Scotland (August), then in England (September). We evaluated how the UK’s published population-level reductions in RSV-related hospitalizations correspond to previously reported vaccine effectiveness (VE) from real-world VE studies.

**Figure:**
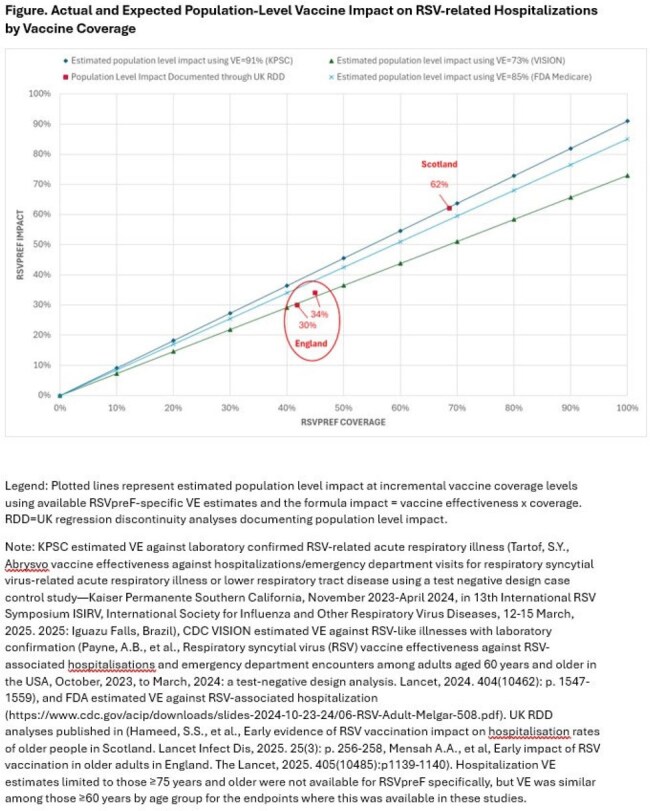
Legend: Plotted lines represent estimated population level impact at incremental vaccine coverage levels using available RSVpreF-specific VE estimates and the formula impact = vaccine effectiveness x coverage. RDD=UK regression discontinuity analyses documenting population level impact. Note: KPSC estimated VE against laboratory confirmed RSV-related acute respiratory illness (Tartof, S.Y., Abrysvo vaccine effectiveness against hospitalizations/emergency department visits for respiratory syncytial virus-related acute respiratory illness or lower respiratory tract disease using a test negative design case control study—Kaiser Permanente Southern California, November 2023-April 2024, in 13th International RSV Symposium ISIRV, International Society for Influenza and Other Respiratory Virus Diseases, 12-15 March, 2025. 2025: Iguazu Falls, Brazil), CDC VISION estimated VE against RSV-like illnesses with laboratory confirmation (Payne, A.B., et al., Respiratory syncytial virus (RSV) vaccine effectiveness against RSV-associated hospitalisations and emergency department encounters among adults aged 60 years and older in the USA, October, 2023, to March, 2024: a test-negative design analysis. Lancet, 2024. 404(10462): p. 1547-1559), and FDA estimated VE against RSV-associated hospitalization (https://www.cdc.gov/acip/downloads/slides-2024-10-23-24/06-RSV-Adult-Melgar-508.pdf). UK RDD analyses published in (Hameed, S.S., et al., Early evidence of RSV vaccination impact on hospitalisation rates of older people in Scotland. Lancet Infect Dis, 2025. 25(3): p. 256-258, Mensah A.A., et al, Early impact of RSV vaccination in older adults in England. The Lancet, 2025. 405(10485):p1139-1140). Hospitalization VE estimates limited to those ≥75 years and older were not available for RSVpreF specifically, but VE was similar among those ≥60 years by age group for the endpoints where this was available in these studies.

**Figure:**
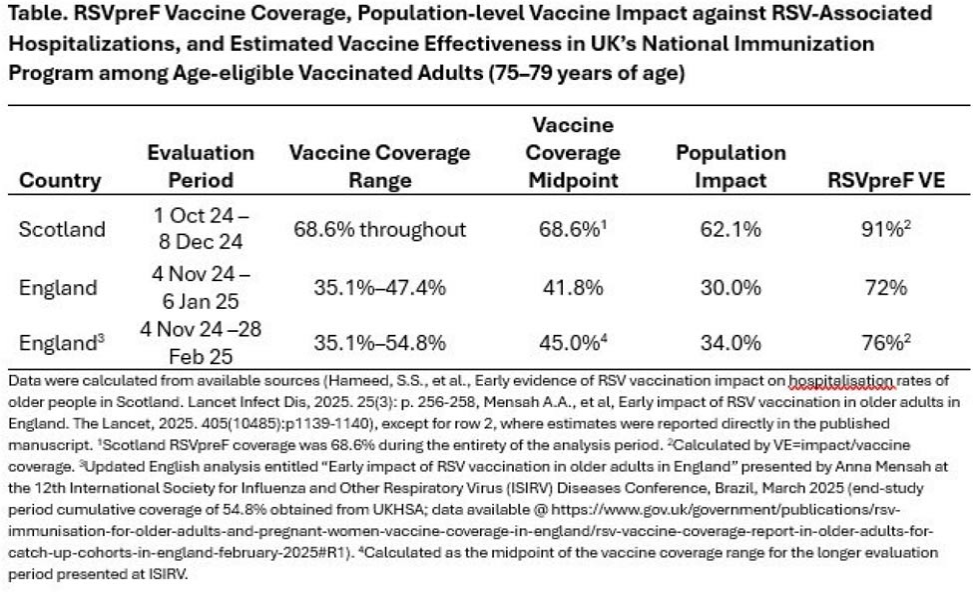
Data were calculated from available sources (Hameed, S.S., et al., Early evidence of RSV vaccination impact on hospitalisation rates of older people in Scotland. Lancet Infect Dis, 2025. 25(3): p. 256-258, Mensah A.A., et al, Early impact of RSV vaccination in older adults in England. The Lancet, 2025. 405(10485):p1139-1140), except for row 2, where estimates were reported directly in the published manuscript. 1Scotland RSVpreF coverage was 68.6% during the entirety of the analysis period. 2Calculated by VE=impact/vaccine coverage. 3Updated English analysis entitled “Early impact of RSV vaccination in older adults in England” presented by Anna Mensah at the 12th International Society for Influenza and Other Respiratory Virus (ISIRV) Diseases Conference, Brazil, March 2025 (end-study period cumulative coverage of 54.8% obtained from UKHSA; data available @ https://www.gov.uk/government/publications/rsv-immunisation-for-older-adults-and-pregnant-women-vaccine-coverage-in-england/rsv-vaccine-coverage-report-in-older-adults-for-catch-up-cohorts-in-england-february-2025#R1). 4Calculated as the midpoint of the vaccine coverage range for the longer evaluation period presented at ISIRV.

**Methods:**

We identified available estimates for RSVpreF VE and population-level impact data. Using the formula impact=VE*uptake, we graphed expected vaccine impact across all potential vaccine coverage values based on available VE data against RSV-related hospitalization and published impact results. We also estimated VE based on published impact results.

**Results:**

In Scotland and England, RSV-related hospitalizations decreased 62% and 30–34%, respectively, based on regression discontinuity methods. We identified three real-world RSVpreF-specific VE estimates against RSV-related hospitalizations (all from US): 73% (CDC VISION), 85% (FDA Medicare), and 91% (Kaiser Permanente Southern California). Several factors likely contributed to variability in these VEs (e.g., duration of post-vaccination follow-up, use of specimen salvage, vaccine exposure data quality, and outcome definitions). Impact measured in the UK closely aligned with expected results estimated from VE studies (Figure). VEs calculated from impact estimates varied: 91% [Scotland] to 72–76% [England] (Table). Stability in vaccine coverage across evaluation periods may contribute to this variability; Scotland’s coverage was ∼69% throughout, while England’s rose from 35–47%.

**Conclusion:**

Early impact results from UK’s RSVpreF vaccination program among adults aged 75–79 are consistent with previously reported VE results from the US. The initial public health impact of this vaccination program in preventing RSV-related hospitalizations is already substantial and could increase with higher vaccine uptake, expansion to other at-risk populations, and inclusion of the expected multiple seasons of protection from vaccination. Protection of older adults from severe RSV disease is off to a promising start.

**Disclosures:**

Negar Aliabadi, MD, MS, Pfizer: i am an employee|Pfizer: Stocks/Bonds (Public Company) Qing Liu, M.S., Pfizer Inc.: I am Pfizer employee and hold Pfizer stocks|Pfizer Inc.: Stocks/Bonds (Public Company) Carmen Hockey, MBChB MSc, Pfizer Ltd: Stocks/Bonds (Private Company) Andrew Vyse, Ph.D., Pfizer UK Ltd: Ownership Interest|Pfizer UK Ltd: Stocks/Bonds (Private Company) Gillian Ellsbury, MD, Pfizer Ltd: Stocks/Bonds (Public Company) Luis Jodar, PhD, Pfizer Inc: Stocks/Bonds (Public Company) Elizabeth Begier, MD, M.P.H., Pfizer: I am an employee.|Pfizer: Stocks/Bonds (Public Company)

